# Effects of Pre-Exhausting the Biceps Brachii Muscle on the Performance of the Front Lat Pull-Down Exercise Using Different Handgrip Positions

**DOI:** 10.2478/hukin-2014-0070

**Published:** 2014-10-10

**Authors:** José Vilaça-Alves, Lurdes Geraldes, Helder M. Fernandes, Luís Vaz, Renato Farjalla, Francisco Saavedra, Victor M. Reis

**Affiliations:** 1 University of Trás-os-Montes and Alto Douro (UTAD), Vila Real, Portugal.; 2 Research Center for Sport, Health and Human Development (CIDESD), Vila Real, Portugal.; 3 Estácio de Sá University, Petrópolis, Rio de Janeiro, Brazil.

**Keywords:** resistance training, front lat pull-down, handgrip width, biceps brachii, perceived exertion

## Abstract

The aim of the present study was to investigate the effects of pre-exhaustion (PE) of the biceps brachii muscle (BB) on the number of repetitions and the rate of perceived exertion (RPE) in the front lat pull-down (FLPD) using different handgrip positions. Additionally, the effect of sex and its interaction with performance and the RPE were also examined. The participants were 19 healthy subjects: 8 men (age: 27.13±2.85 years; body height: 180.63±6.65 cm; body mass: 82.05±8.92 kg; and body fat: 14.67±6.09%); and 11 women (age: 28.81±3.68 years; body height: 162.91±6.51 cm; body mass 59.63±6.47 kg; and body fat: 24.11±4.33%). The number of repetitions and the RPE in the FLPD exercise with different handgrip positions, with and without PE of the BB, was documented. The following main significant effects were seen: i) PE of the BB decreased the number of repetitions (p<0.001) and increased the RPE (p<0.001); ii) the narrow handgrip width elicited a higher RPE (p<0.001) and iii) women performed fewer repetitions than men in all FLPD exercise variations (p=0.023). Significant interactions were also observed between: i) PE or sex and the RPE (p=0.024); and ii) PE or handgrip width and the number of repetitions (p<0.001). In conclusion, PE of the BB promotes a decreased performance in the FLPD exercise along with a greater RPE, especially when using a narrow handgrip position.

## Introduction

An appropriate and efficient combination of resistance training (RT) variables is crucial to the subsequent adaptations and the achievement of a specific goal. Possible RT variations include changing: the number of sets, repetitions, and exercises; the rest interval between sets and exercises, the number of training days within a week; the load used; the type of muscle contraction; the cadence of movement; the choice of specific exercises; and the execution order of exercises (Fleck and Kraemer, 1996). Numerous studies have focused on the influence of exercise order on muscle performance (Sforzo and Touey, 2006; Simão et al., 2005; Spreuwenberg et al., 2006; Romano et al., 2013). Based on aforementioned research it seems that a larger amount of work can be performed in exercises at the beginning of the training session, both in exercises that involve the upper limbs (Simão et al., 2005) and those that involve the lower limbs (Simão et al., 2005; Spreuwenberg et al., 2006).

Pre-exhaustion (PE) is a RT method of combining two exercises, in which a single-joint exercise performed exhaustively is followed by a multi-joint exercise. The purpose of PE of the smaller muscle is to provide lower involvement of this muscle in the subsequent multi-joint exercise, thereby enabling greater participation of other muscles. Augustsson et al. (2003) examined the performance in the squat exercise when preceded by leg extension exercise and found decreased muscle activity and a reduction in strength when performing the squat exercise after PE. This finding may be explained by the fact that the quadriceps femoris muscle group, a major agonist in both exercises, had been pre-exhausted.

One popular upper body RT exercise is the lat pull-down (LPD) involving the intervention of several muscle groups: the latissimus dorsi, the rhomboids, the middle trapezius, and the biceps brachii (BB). The LPD exercise can be performed with different variations: the bar can be pulled to the upper chest (FLPD) or to the back of the neck; the handgrip can vary in width (wide vs. narrow); and the handgrip can be in pronation or supination. Several authors have addressed the effect of the use of such variations on the involvement of different muscle groups that intervene in the exercise. Sperandei et al. (2009) compared front vs. behind the neck LPD exercise with a V bar and concluded that the safest way to perform the exercise was to pull the bar to the upper chest (FLPD exercise). Lustk et al. (2010) observed that there was greater activation of the latissimus dorsi muscle during the FLPD exercise with hands pronated, regardless of the handgrip distance. In addition, with the hands pronated, the BB was the most elicited upper arm muscle group when a handgrip of shoulder width was used.

With respect to possible sex differences, studies have shown that males have significantly higher muscle strength than females, especially in the upper body and in the muscle groups that present a higher percentage of fat-free mass (Hubal et al., 2005; Staron et al., 1994). However, a meta-analysis presented by Rhea and Alderman (2004) reported similar responses in muscle strength levels between sexes when only fat-free mass was considered. According to Lemmer et al. (2007), the differences between sexes in maximum strength vary at different body sites. For example, females present a slightly larger relative strength in the lower body when compared with males. Kell (2011) observed a better adaptation by female subjects to a RT program, as compared with males. Salvadora et al. (2009) observed a significant decrease in the fatigue rate of female subjects when compared with males.

Although RT instructors tend to use PE training techniques as a method for eliciting greater muscle activation during the subsequent multi-joint exercises, its effects on all types of upper body strength exercises is not yet well understood. Therefore, it is relevant to study whether PE of the BB muscle group influences the number of repetitions and the rate of perceived exertion (RPE) of the FLPD exercise when using different handgrip distances. Therefore, the aim of the present study was to investigate the effects of pre-exhaustion (PE) of the biceps brachii muscle (BB) on the number of repetitions and the rate of perceived exertion (RPE) in the front lat pull-down (FLPD) using different handgrip positions.

## Material and Methods

### Participants

Nineteen healthy, physically active Caucasian subjects (8 men and 11 women) volunteered to participate in this study. Table 1 provides the characteristics of the participants by sex. Every selected participant had been engaged in physical activity (comprising resistance training) for at least 3 days per week over the previous 6 months. Participants were informed about the possible risks or discomfort involved in the experiment and provided written informed consent to participate in the study. The procedures were designed according to the Helsinki Declaration and were approved by the Ethics Committee of the University of Trás-os-Montes & Alto Douro.

### Procedures and Measures

All participants were evaluated in a total of six sessions on non-consecutive days. Session 1 was dedicated to the measurement of body mass, height, body fat, and 1-repetition maximum tests (1RM). Anthropometric measures were recorded in light clothing using a portable stadiometer (Sanny ES 2030, Physical Nutri, Araraquara, SP, Brazil) with accuracy of 0.1 cm and a portable scale (Seca, Cirencester, UK) with accuracy of 0.1 kg. Skinfold measurements were performed by a trained and experienced technician, using a skinfold calliper (Sanny AD1010, Physical Nutri, Araraquara, SP, Brazil) according to standardized procedures (ACSM, 2010). The measurements were made in triplicate, and their means were used in further calculation. Body fat was estimated from measurements of skinfold thickness by using the generalized skinfold equations from Jackson and Pollock (1978) and Jackson et al. (1980). In session 2, the 1RM was reevaluated. In sessions 3 and 4, the number of repetitions and RPE values in the FLPD with a wide grip (FLPD-WG) or in FLPD with a normal grip (FLPD-NG) positions were measured. In session 3 the choice of the exercise was randomly selected, and in session 4 the FLPD was performed with a different grip than in the previous session. In sessions 5 and 6, the same variables and exercises of sessions 3 and 4 were measured and performed, respectively, after PE of the *biceps brachii* muscle. The PE was obtained by using the arm curl exercise with a barbell (BC). All resistance exercises were performed with 70% of the 1RM load with an up-down cadence of 60 beats/min (30 repetitions per minute) controlled by an electronic metronome (Korg MA-30, Korg, Melville, New York, USA). Three sets of 10 repetitions of the BC exercise were used to promote pre-exhaustion with a rest interval of 90 s between sets. The BC exercise was performed using an underhand grip at shoulder width. An interval of 72 hours between sessions was assured. In addition, all subjects were instructed to refrain from any strenuous physical activity (e.g., cycling, running, weight lifting and recreational activities) during the experimental period.

### Strength Testing

The 1RM testing protocol was described by Kraemer and Fry (1995). The participants performed a 1RM familiarization protocol in all strength exercises performed in the first four sessions (Levinger et al., 2009), with an emphasis on the specific technique and breathing rhythm for lifting. An electronic metronome was used for pacing to control exercise rhythm. The heaviest load achieved was considered the 1RM load. To minimize the error during the 1RM tests, the following strategies were adopted (Simão et al., 2007): a) standardized instructions concerning the testing procedures were given to participants before the test; b) participants received standardized instructions on exercise technique; c) the 60 beats/min cadence was maintained; and d) standard verbal encouragement was provided during the testing procedure. The 1RM was determined in 5 attempts with a rest interval of 5 minutes between them.

### Handgrip Width, Number of Repetitions and Rate of Perceived Exertion

The FLPDs with a narrow and wide grip, with and without PE, were performed following the procedures suggested by Graham (2003) differing only by the handgrip width. The FLPDNG was performed with the handle grip at shoulder width and with FLPD-WG at the downward bend of the bar. The subjects were instructed not to pause at each metronome beep. On the FLPD-NG and on the FLPD-WG, the number of repetitions performed was measured by counting the number of correct executions of the movement when the bar was in the start position. If the subjects did not maintain the cadence or the correct execution for two repetitions, the counting stopped and the number of repetitions was registered. At the last repetition of each exercise, the RPE was recorded by showing the OMNI-RES scale. The OMNI-RES scale was always visible for the participant, and a previous familiarization period was implemented according to the procedures suggested by Lagally and Robertson (2006).

### Statistical analysis

The data were processed using SPSS 21.0 for Mac and presented as mean and standard deviation. Shapiro-Wilk, Levene and Mauchly’s tests were used in order to check the normality, homogeneity and sphericity, respectively, of the sample’s data variances. The intraclass correlation coefficient (ICC) was used to measure the repeatability on the strength tests. A 2 (groups) x 2 (with and without PE) x 2 (handgrips width) repeated measures ANOVA was performed. Partial eta-squared values **(ηp^2^)** were reported as measures of effect size, with values higher than 0.01, 0.06 and 0.14 representing small, medium, and large effects, respectively (Cohen, 1988). The level of significance was set at p<0.05.

## Results

Excellent day-to-day 1RM reliability was shown for the BC and FLPD exercises, namely, BC (ICC=0.99), FLPWG (ICC=0.99), and FLPDNG (ICC=0.98).

Table 2 presents the number of exercise repetitions and the RPE according to different handgrip conditions and sex.

The main negative effect of the BB EE on the number of repetitions (*F*=101.22; *p*<0.001; η_p_^2^=0.86), on the RPE (F=66.92; p<0.001; η_p_^2^= 0.80) and on the FLPD performance with the two handgrips width was observed in both sexes. The narrow handgrip also negatively influenced the RPE on the FLPD performance with and without pre-exhaustion in both sexes (*F*=105.78; *p*<0.001; η_p_^2^=0.86). A lower number of repetitions in all FLPD execution variations was observed (*F*=6.21; *p*=0.023; η_p_^2^=0.27) in women. Furthermore, an interaction between PE and sex on the RPE (*F*=6.15; *p*=0.024; η_p_^2^=0.27) was also noted in the FLPD-NG, with women showing a higher increment in the RPE after PE. PE also showed an interaction with the grip width in the FLPD execution. More specifically, the FLPD-NG condition showed a reduced number of repetitions (*F*=20.04; *p*<0.001; η_p_^2^=0.54) when compared with the FLPD-WG condition (both with PE).

## Discussion

An important finding of the present study was that a significant decrease was observed, in both sexes, in the performance of the FLPD-WG and FLPD-NG exercises after PE. These data are in line with the study by Augustsson et al. (2003) who documented a decrease in the number of repetitions with the leg press exercise after performing PE with leg extension exercise. These authors also observed a decrease in the electromyography signal of the rectus femoris and the vastus lateralis muscle, showing that the most elicited muscle groups responded to PE by decreasing their ability to create tension. Gentil et al. (2007) and Brennecke et al. (2009) also observed that PE caused a decrease in the electromyography signal in the pre-exhausted muscle group, namely the pectoralis major muscle group during the horizontal chest press exercise (pre-exhausted with the peck-deck exercise).

Regarding handgrip width, a significant decrease in the number of repetitions between FLPD-WG and the FLPD-NG was observed only when the exercises were performed following PE of the BB. Handa et al. (2005), using surface electromyography, observed lower activation of the BB when a wide handgrip width was used with the FLPD exercise. Signorile et al. (2002) reported greater intervention of the BB muscle during the FLPD when the handgrip width was narrow. Since the exercise used for pre-exhaustion in this study was the biceps curl and a major muscle in this movement is the BB, the decrease in the number of repetitions with the FLPD-NG when compared to the FLPD-WG may be explained by the considerable involvement of the BB in the latter. However, Lusk et al. (2010) did not observe significant changes in the electromyography signal of the latissimus dorsi, middle trapezius and BB muscle groups with wide or narrow handgrips. The width of the narrow grip condition in the two studies was much different, and this may account for the different results that were reported.

No significant differences were observed in the number of repetitions in the FLPD performed by males vs. females, regardless of the grip used. Likewise, in a study by Johnson et al. (2009), no differences between sexes were observed in the same exercise performed with a wide handgrip, in which the BB muscle is probably less activated (Brennecke et al., 2009). Lemmer et al. (2007) suggested that the BB muscle and the upper body muscle groups in general, had a smaller capacity to alter the production of maximum strength in females as compared to males. Moreover, in elderly women a significantly inferior capacity for strength production was observed with the upper body muscles when compared with their male peers (Wiacek and Zubrzycki, 2010). On the other hand, Kell (2011) observed a greater increase in strength in females compared to males. However, the female participants of the Kell’s (2011) study were untrained while male subjects were trained. In fact, training progression is greater in trained subjects in comparison with untrained ones.

Regarding the RPE, no significant differences were observed between sexes in either FLPD variations analysed. However, PE and the handgrip width influenced the RPE, with higher values of the RPE observed in the FLPD-NG condition. The RPE was influenced by the interaction between PE and sex, with women showing a higher increment of RPE values in the FLPD-NG when compared with the values of no PE. This may be related to the fact that the narrow handgrip width fosters a superior recruitment of minor muscle groups (e.g., the BB) and may be more easily fatigued, resulting in higher RPE values by females. This argument corroborates the findings of Lemmer et al. (2007) who indicated that women had a reduced capacity of maximum strength production in BB muscle and consequently presented a greater RPE in efforts that required greater intervention by this muscle group.

## Conclusions

Based on the present results, we may conclude that within both sexes the pre-exhaustion of the biceps brachii muscle can promote greater fatigue and a higher RPE in the subsequent execution of the FLPD. The FLPD with narrow handgrip width also appears to be influenced negatively by PE in the number of repetitions and the RPE in both sexes. Nevertheless, a higher RPE was observed in the females when performing the FLPD-NG after PE. Future studies should be carried out using electromyography analysis in order to better understand muscle recruitment differences during the FLPD exercise performed after pre-exhaustion and using different handgrips widths.

## Figures and Tables

**Table 1:** Characteristics of the participants

	**Men** (n=8)	**Women** (n=11)
Age (years)	27.13±2.85	28.82±3.68
Body height (cm)	180.63±6.65	162.91±6.52
Body mass (kg)	82.05±8.92	59.63±6.47
Estimated body fat (%)	14.67±6.09	24.11±4.33
1RM FLPD-WG (kg)	81.25±18.40	44.36±6.00
1RM FLPD-NG (kg)	85.13±21.66	49.27±5.76
1RM BC (kg)	38.50±7.76	18.36±3.32

Values are given as means ± SD; FLPD-WG – Front lat pull-down exercise with wide grip; FLPD-NG – Front lat pull-down with narrow grip; BC – Biceps curl; 1RM – one repetition maximum

**Table 2:** Number of repetitions (rep) and rate of perceived exertion (RPE) in the front lat pull-down exercise (FLPD) using different handgrip positions.

	**Men** (n=8)		**Women** (n=11)	
	Without PE	With PE	Without PE	With PE
FLPD-WG (rep)	13.63±1.51	10.75±2.66	12.18±1.78	9.91±1.70
FLPD-NG (rep)	14.50±0.53	9.38±1.85	11.36±2.62	8.09±1.87
RPE FLPD-WG	7.88±0.35	8.75±0.46	7.64±0.67	8.55±0.52
RPE FLPD-NG	9.50±0.54	9.75±0.46	9.18±0.41	9.73±0.91

Values are given as means ± SD; FLPD-WG – Front lat pull-down exercise with wide grip; FLPD-NG – Front lat pull-down with narrow grip

## References

[b1-jhk-42-157] American College of Sports Medicine (2010). ACSM's guidelines for exercise testing and prescription.

[b2-jhk-42-157] Augustsson J, Thomee R, Hornstedt P, Lindblom J, Karlsson J, Grimby G (2003). Effect of pre-exhaustion exercise on lower-extremity muscle activation during a leg press exercise. J Strength Cond Res.

[b3-jhk-42-157] Brennecke A, Guimarães T, Leone R, Cadarci M, Mochizuki L, Simão R, Amadio AC, Serrão JC (2009). Neuromuscle activity during bench press exercise performed with and without the preexhaustion method. J Strength Cond Res.

[b4-jhk-42-157] Cohen J (1988). Statistical Power Analysis for the Behavioral Sciences.

[b5-jhk-42-157] Fleck SJ, Kraemer WJ (2006). Designing resistance training programs.

[b6-jhk-42-157] Gentil P, Oliveira A, Rocha Júnior V, Do Carmo J, Bottaro M (2007). Effects of exercise order on upper-body muscle activation and exercise performance. J Strength Cond Res.

[b7-jhk-42-157] Graham J (2003). Front Lat Pulldown. Strength Cond J.

[b8-jhk-42-157] Handa T, Kato H, Hasegawa S, Okada J, Kato K (2005). Comparative electromyographical investigation of the biceps brachii, latissimus dorsi, and trapezius muscles during five pull exercises. Jap J Phys Fit Sports Med.

[b9-jhk-42-157] Hubal MJ, Gorddish-Dressman H, Thompson PD, Price TB, Hoffman EP, Angelopoulos TJ, Gordon PM, Moyna NM, Pescatello LS, Visich PS, Zoeller RF, Seip RL, Clarkson PM (2005). Variability in muscle size and strength gain after unilateral resistance training. Med Sci Sports Exer.

[b10-jhk-42-157] Jackson AS, Pollock ML, Ward A (1980). Generalized equations for predicting body density of women. Med Sci Sports Exerc.

[b11-jhk-42-157] Jackson AS, Pollock ML (1978). Generalized equations for predicting body density of men. Br J Nutr.

[b12-jhk-42-157] Johnson D, Lynch J, Nash K, Cygan J, Mayhew J (2009). Relationship of lat-pull repetitions and pull-ups to maximal lat- pull and pull-up strength in men and women. J Strength Cond Res.

[b13-jhk-42-157] Kell RT (2011). The influence of periodized resistance training on strength changes in men and women. J Strength Cond Res.

[b14-jhk-42-157] Kraemer W, Fry A, Maud PJ, Foster C (1995). Strength testing: Development and evaluation of methodology. Physiological Assessment of Human Fitness.

[b15-jhk-42-157] Lagally K, Robertson R (2006). Construct validity of the OMNI resistance exercise scale. J Strength Cond Res.

[b16-jhk-42-157] Lemmer T, Martel G, Hurlbut D, Hurley B (2007). Age and sex differentially affect regional changes in 1 repetition maximum strength. J Strength Cond Res.

[b17-jhk-42-157] Levinger I, Goodman C, Hare DL, Jerums G, Toia D, Selig S (2009). The reliability of the 1RM strength test for untrained middle-aged individuals. J Sci Med Sport.

[b18-jhk-42-157] Lusk S, Hale B, Russell D (2010). Grip width and forearm orientation effects on muscle activity during the lat pull-down. J Strength Cond Res.

[b19-jhk-42-157] Rhea MR, Alderman BL (2004). A meta-analysis of periodized versus nonperiodized strength and power training programs. Res Q Exerc Sport.

[b20-jhk-42-157] Romano N, Vilaça-Alves J, Fernandes HM, Saavedra F, Paz G, Miranda H, Simão R, Novaes J, Reis V (2013). Effects of Resistance Exercise Order on the Number of Repetitions Performed to Failure and Perceived Exertion in Untrained Young Males. J Hum Kinet.

[b21-jhk-42-157] Salvadora E, Diasc M, Gurjão A, Avelara A, Pintod L, Cyrinoa E (2009). Effect of eight weeks of strength training on fatigue resistance in men and women. Isokinet Exerc Sci.

[b22-jhk-42-157] Sforzo A, Touey P (2006). Manipulating exercise order affects muscle performance during a resistance exercise training session. J Strength Cond Res.

[b23-jhk-42-157] Signorile J, Zink A, Szwed S (2002). A comparative electromyographical investigation of muscle utilization patterns using various hand positions during the lat-pull-down. J Strength Cond Res.

[b24-jhk-42-157] Simão R, Farinatti P, Polito M, Maior A, Fleck S (2005). Influence of exercise order on the number of repetitions performed and perceived exertion during resistance exercises. J Strength Cond Res.

[b25-jhk-42-157] Simão R, Farinatti PTV, Polito MD, Viveiros L, Fleck SJ (2007). Influence of exercise order on the number of repetitions performed and perceived exertion during resistance exercise in women. J Strength Cond Res.

[b26-jhk-42-157] Sperandei S, Barros M, Silveira-Júnior P, Oliveira C (2009). Electromyographic analysis of three different types of lat pull-down. J Strength Cond Res.

[b27-jhk-42-157] Spreuwenberg LPB, Kraemer WJ, Spiering BA, Volek JS, Hatfield DL, Silvestre R, Vingren JL, Fragala MS, Häkkinen K, Newton RU, Maresh CM, Fleck SJ (2006). Influence of exercise order in a resistance-training exercise session. J Strength Cond Res.

[b28-jhk-42-157] Staron RS, Karapondo DL, Kraemer WJ, Fry AC, Gordon SE, Falkel JE, Hagerman FC, Hikida RS (1994). Skeletal muscle adaptations during early phase of heavy resistance training in men and women. J App Physiol.

[b29-jhk-42-157] Wiacek M, Zubrzycki I (2010). The age-dependent divergence of strength and coordinating parameters among men and women: The cross-sectional studies. Arch Gerontol Geriatr.

